# Age-related differences in factors associated with the underuse of recommended medications in acute coronary syndrome patients at least one year after hospital discharge

**DOI:** 10.1186/1471-2261-14-127

**Published:** 2014-09-24

**Authors:** Hong Jin, Chengchun Tang, Qin Wei, Long Chen, Qin Sun, Genshan Ma, Naifeng Liu

**Affiliations:** Department of Cardiology, Zhongda Hospital, Medical School of Southeast University, 210009 Nanjing, Jiangsu China

**Keywords:** Medications, Secondary prevention, Age factors, Aged, Coronary heart disease

## Abstract

**Background:**

Few studies have evaluated age-related predictors associated with the underuse of medications in patients with coronary heart disease (CHD). The objective of this study was to identify age-related differences in the factors associated with the underuse of recommended medications in patients diagnosed with acute coronary syndrome (ACS).

**Methods:**

From August 2009 to April 2011, we recruited 469 consecutive ACS patients from a cardiac center at a university hospital. We divided the patients into older (65 years of age and older, n = 202) and younger groups (younger than 65 years of age, n = 267). Data on socio-demographic characteristics, depressive symptoms, and medication use were obtained from a telephone survey administered 18 to 24 months after hospital discharge. Additionally, we asked the patients to provide reasons for not taking their medications.

**Results:**

A significantly increased underuse of medication was noted in older patients compared with younger patients, including aspirin (24.8% vs. 37.1%, p = 0.005), beta-blockers (20.3% vs. 34.8%, p = 0.001), ACE inhibitor/angiotensin receptor blockers (27.2% vs. 36.7%, p = 0.030), and statins (21.8% vs. 29.6%, p = 0.005). Among older patients, the factors associated with the underuse of medications included low education level (odds ratio [OR], 3.93), greater number of comorbidities (OR, 1.64), and total number of discharge medications (OR, 1.31). The reasons provided by older patients for not taking medication included the fact that the medication was considered to be non-essential and the large number of medications. Among younger patients, low income (OR, 3.97) and depression (OR, 2.62) were predictors for underuse of medication, and the reasons provided for not taking medications included high costs and the fear of adverse effects.

**Conclusions:**

At least one year after ACS hospital discharge, the underuse of recommended medications is related to low education level, comorbidities, and the total number of discharge medications in elderly patients, whereas underuse in younger patients is associated with low income and depression. The disparities related to these different predictors may have implications for age-related interventions targeting secondary preventions in CHD patients to improve their use of medication.

## Background

Coronary heart disease (CHD) is the leading cause of morbidity and mortality in China [1–3]. Urbanization, industrialization, and population aging have resulted in a rapid and significant increase in the prevalence of CHD in recent decades [1]. Unstable angina (USA), Non-ST segment elevation myocardial infarction (NSTEMI), and ST-segment elevation myocardial infarction (STEMI) are common manifestations of acute coronary syndrome (ACS) and are major causes of hospitalizations [4–6]. Conversely, this rate has decreased over the last 3 decades because of the attention given to coronary risk factors and improvements in clinical management [5, 6]. Therefore, efforts should be refocused on secondary prevention. The secondary prevention of CHD involves managing both lifestyle factors and physiologic parameters, often with medications. It is well accepted that adequate treatments with multiple evidence-based medications and vigorous control of major risk factors in CHD patients are cost-effective secondary strategies [7, 8]. More than 40% of the recent decline in CHD mortality has been attributed to evidence-based medications [9]. The recommended medications for patients who have previously experienced an ACS episode include the concurrent use of lipid-lowering agents, antiplatelet medications, beta-blockers, and angiotensin-converting enzyme inhibitors (ACEI) or angiotensin II receptor blockers (ARB) [10, 11].

Despite the large amount of evidence supporting the use of recommended medications and the development of public policies, which have led to significant improvements in ACS management and its consequences in recent years, the underuse of optimal cardioprotective medications is prevalent among CHD outpatients and is associated with a broad range of adverse outcomes, including all-cause and cardiovascular mortality, cardiovascular hospitalizations, and revascularization procedures [12–14]. However, in many developed countries, the rate of recommended medication use is reportedly low [9, 13, 15], particularly for older patients [16]. Several studies have suggested that age-related differences in the use of secondary medical preventions are a widespread phenomenon among CHD patients and that older patients are less likely to take recommended medications [16–18]. However, few studies have attempted to explain the age-related differences in the underuse of these secondary prevention medications based on socio-demographic factors and clinical characteristics or to evaluate the differences in terms of the reasons why patients did not take their medications.

We performed a long-term observational study with the following objectives: 1) to better characterize the underuse of recommended medications for secondary prevention in Chinese ACS patients at least one year after hospital discharge; 2) to determine whether age is associated with the underuse of medications in these high-risk CHD patients; and 3) to identify differences in the factors associated with the underuse of recommended medications between age groups. Additionally, we explored the patients’ reasons for not taking their recommended medications.

## Methods

### Study population

From August 2009 to April 2011, consecutive ACS patients were selected from a cardiac center at a university hospital, located in Nanjing. Diagnosis of ACS is based upon symptom history, clinical presentation, electrocardiogram ST-segment changes, and enzyme elevation. All participants provided full written informed consent, and the study was approved by the Committee of Clinical Investigation at Southeast University School of Medicine. The following exclusion criteria were applied: age older than 85 years, age younger than 18 years, severe hearing impairment, unintelligible speech, lack of cooperation, delirium or severe dementia, illness that interfered with participation, overt psychiatric illness, administration of all medications by a caregiver, and no telephone number. Given that the purpose of our study was to assess the underuse of recommended medications in ACS patients at least one year after discharge, patients were also excluded if they suffered from an ACS episode within 12 months and if they participated in other clinical trials.

**Figure 1:**
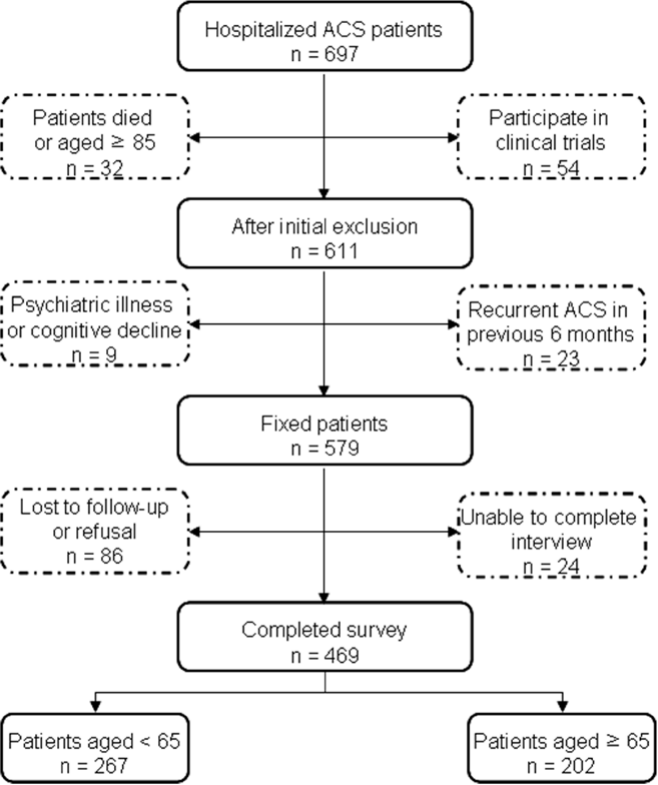
**Recruitment flowchart.** ACS, acute coronary syndrome.

In total, 697 eligible patients were identified during the study period. Of these patients, 118 were excluded; 17 patients died before the survey was administered; 15 patients were older than 85 years of age; 54 patients participated in other clinical trials; 9 patients had a psychiatric illness or cognitive decline, and 23 patients were hospitalized for a recurrent acute coronary event during the 12-month period before the survey was administered. Of the 579 patients included in the study, 63 patients were unavailable for follow-up, and 23 patients did not complete the telephone review. An additional 24 patients refused to respond to a telephone survey. Hence, a total of 469 patients were included in this analysis (Figure 1). We use 65 years of age and older as the definition of an older individual because most countries have accepted this definition of old age. With regard to anthropometric, socioeconomic, and clinical characteristics, no statistically significant differences were noted between the responders and non-responders.

### Design and data collection

While still hospitalized following their procedures, the patients were approached by a researcher who assessed their eligibility for study enrollment. Baseline medications were collected using medical charts and electronic medical records. The collected data included patient demographics, past medical history, clinical characteristics, comorbidities, inpatient treatment, medications prescribed at the time of discharge, and in-hospital outcomes. The recommended medications prescribed at the time of discharge, including aspirin, beta-blockers, statins, and ACEI/ARB, were obtained from the medical records. ACEI and ARB were grouped as a single variable because they are both used in the treatment of hypertension and congestive heart failure, and ARB is generally administered to patients who are intolerant of ACEI. The variable “total number of discharge medications” was the sum of all chronic medications mentioned by the patient at the time of discharge from the hospital.

During the study period, follow-up information was obtained from patients via telephone surveys conducted by trained interviewers approximately 18 to 24 months after discharge. The purpose of this survey was to document the occurrence of secondary cardiovascular events, hospitalizations, and scheduled or unscheduled outpatient cardiologist visits between the time of discharge and the telephone survey. We also assessed current medication use and depressive symptoms. At least 3 attempts to contact patients were made at various times of the day. For the purpose of this analysis, the length of time in days from the date of discharge to the date of telephone contact (interim period time) was taken as the number of months post discharge.

The telephone questionnaire focused on the following points. The first section included patient baseline characteristics, such as age, sex, educational level, marital status, income, living situation, smoking, alcohol use, medical history, and depressive symptoms. To evaluate the presence and severity of depressive symptoms, we administered the well-validated 9-item Patient Health Questionnaire (PHQ) [19]. Higher scores indicated more severe depressive symptoms. Our primary predictor variable was a single question regarding the treatment status of aspirin, statins, beta-blockers, and ACEI or ARB. Information regarding medication use was obtained by asking the patients to provide their medication bottles and to respond to the question, “What medications do you take now?” If the patients replied, “none,” the reasons for not taking the medications were explored. To perform this assessment, the patients were asked, “Which one of the following is the reason for not taking it?” The patients were prompted with possible options, including being unable to afford the cost of the medications, considering the medications to be non-essential, having been prescribed too many medications, being concerned about adverse effects, and suffering side effects. The patients were asked to choose their reasons for not taking the medications. More than one reason could be recorded.

### Statistical analyses

The data are presented as the mean ± standard deviation for normally distributed data or as the median (interquartile range) for skewed continuous variables. We conducted comparisons of continuous variables using Student’s *t*-test or the Mann–Whitney U test. The chi-squared test was used to compare categorical variables between the two groups. Associations between variables of interest and the underuse of medication were assessed using logistic regression. Significant univariate predictors were included in multivariate logistic regression analysis. We included the following 9 covariates in the model: age, gender, education, insurance, income, comorbidities, PHQ scores, number of medications at the time of discharge, and outpatient cardiologist visit from the time of discharge. We performed statistical analyses using SPSS software 16.0 (SPSS Inc., Chicago, IL, USA). All tests were two-sided. We considered p < 0.05 to be statistically significant.

## Results

Comparison of the anthropometric, socioeconomic, and clinical characteristics of patients in the two age groups is presented in Table 1. The older patients were more likely to live alone and were less likely to have completed senior high school or higher. Compared with the younger group, employment in the older age group was significantly lower. Established comorbidities (congestive heart failure, diabetes mellitus, dyslipidemia, stroke, chronic renal failure, and chronic obstructive pulmonary disease) were more prevalent in the older age group. Compared with the older group, the younger patients had significantly higher PHQ scores. The number of discharge medications prescribed to older patients was greater than that prescribed to younger patients. The younger patients were more likely to see physicians on a regular basis compared with older patients. No between-group differences were observed in terms of marital status, insurance, smoking, number of diseased vessels, management strategies, and rehospitalization from the time of discharge.

**Table 1:** **Characteristics of the study patients based on age group**

	Overall (n = 469)	65 years of age and older (n = 202)	Younger than 65 years of age (n = 267)	p
Age, years	62.4 ± 9.7	71 ± 5	56 ± 6.9	< 0.001
Gender, female, n (%)	142 (30.3)	67 (33.2)	75 (28.1)	0.264
Married	442 (94.2)	187 (92.6)	255 (95.5)	0.229
Educational level				
Junior high school or lower	166 (35.4)	91 (45.0)	75 (28.1)	< 0.001
Senior high school	198 (42.2)	86 (42.6)	112 (41.9)	
University/college	105 (22.4)	25 (12.4)	80 (30.0)	
Manual laborer	48 (10.2)	26 (12.9)	22 (8.2)	0.101
Employed	178 (38)	7 (3.5)	171 (64)	< 0.001
Income (Chinese yuan/month)				
1,200 - 3,000	248 (52.9)	156 (77.2)	92 (34.5)	< 0.001
3,000 - 5,000	166 (35.4)	27 (13.4)	139 (52.1)	
≥ 5,000	55 (11.7)	19 (9.4)	36 (13.5)	
Basic medical insurance^*^	391 (83.4)	162 (80.2)	229 (85.8)	0.109
Live alone	29 (6.2)	20 (9.9)	9 (3.4)	0.004
Currently smoking	111 (23.7)	45 (22.3)	66 (24.7)	0.538
Regular alcohol use	30 (6.4)	12 (5.9)	18 (6.7)	0.726
Depressive symptoms (PHQ score)	4 (2–7)	4 (2–6)	5 (3–7)	0.005
No. of diseased vessels	1 (1–2)	1 (1–2)	1 (1–2)	0.107
Comorbidities				
Congestive heart failure	37 (7.9)	27 (13.4)	10 (3.7)	< 0.001
Diabetes	103 (22.0)	57 (28.2)	46 (17.2)	0.004
Hypertension	262 (55.9)	122 (60.4)	140 (52.4)	0.086
Dyslipidemia	84 (17.9)	49 (24.3)	35 (13.1)	0.002
Cardiac dysrhythmias	31 (6.6)	18 (8.9)	13 (4.9)	0.081
Stroke	27 (5.8)	17 (8.4)	10 (3.7)	0.032
Any previous revascularization	36 (7.7)	21 (10.4)	15 (5.6)	0.054
Chronic renal failure	38 (8.1)	24 (11.9)	14 (5.2)	0.009
COPD	29 (6.2)	19 (9.4)	10 (3.7)	0.012
Gastrointestinal disease	48 (10.2)	27 (13.4)	21 (7.9)	0.052
Others	45 (9.6)	29 (14.4)	16 (6.0)	0.002
In-hospital treatment (PCI/CABG)	444 (94.7)	190 (94.1)	254 (95.1)	0.609
Time between discharge and follow-up survey, months	19.5 ± 2.6	19.3 ± 2.6	19.7 ± 2.6	0.161
Number of outpatient cardiologist visits since hospital discharge	7 (5–9)	6 (4–8)	8 (6–10)	< 0.001
Rehospitalization since discharge^#^	0 (0–1)	0 (0–1)	0 (0–1)	0.092
Medication use at discharge				
Aspirin	445 (94.9)	188 (93.1)	257 (96.5)	0.121
Beta-blocker	420 (89.6)	175 (86.6)	245 (91.8)	0.072
Statin	414 (88.3)	172 (85.1)	242 (90.6)	0.067
ACE inhibitor/ARB	319 (68.0)	135 (66.8)	184 (68.9)	0.632
Number of prescribed medications^§^	6 (5–10)	8 (5–12)	5 (4–7)	< 0.001

Data are represented as the number (%) of patients; *medical insurance provided by the government; STEMI, ST segment elevated myocardial infarction; NSTEMI, non-ST segment elevated myocardial infarction; COPD, chronic obstructive pulmonary disease; CABG, coronary artery bypass graft; PCI, percutaneous coronary intervention; ACEI, angiotensin- converting enzyme; ^#^patient was readmitted for a cardiac catheterization, PCI, stress test, or other causes during the interim follow-up period from hospital discharge to the date of the follow-up survey; ARB, angiotensin receptor blocker; ^§^the number of patients using prescribed medications, including non-recommended medications.

Every patient in the cohort was prescribed at least one recommended medication at hospital discharge. The majority (89.3%) of the population was prescribed three or more recommended medications (e.g., aspirin, beta-blockers, statin, and ACEI/ARB) at hospital discharge (Table 1). The rate of prescribed recommended medications at hospital discharge was similar between the two age groups (Table 1). The number of patients using 10 or more prescribed medications, including non-recommended medications, at the time of discharge was larger in older patients than in younger patients (43.6% of those 65 years of age and older and 12.4% of those younger than 65 years of age) (p < 0.001).

**Figure 2:**
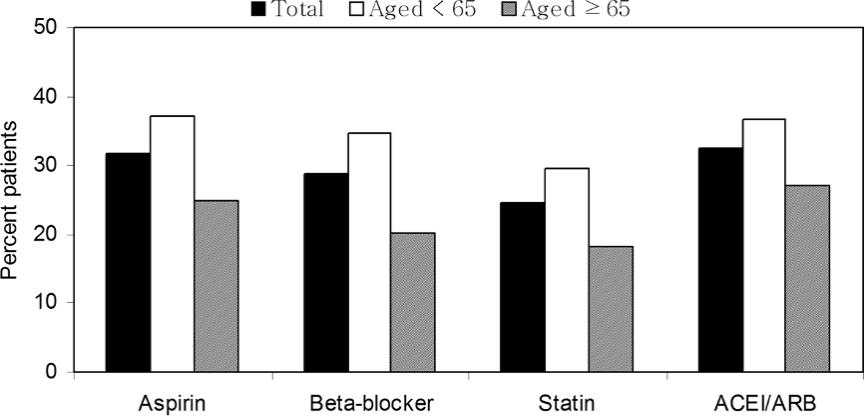
**Percentage of patients taking recommended medications based on age at the time of the survey.** (P-value: aspirin = 0.005, beta-blockers = 0.001, statins = 0.005, angiotensin-converting enzyme inhibitor [ACEI]/angiotensin receptor blocker [ARB] = 0.030, between age groups).

At the time of the follow-up survey, 39.7% patients were not taking any recommended medications (51.5% in the older group and 30.7% in the younger group). Figure 2 shows the rate of recommended medication use for the total number of patients and for each age group at the time of the survey. Overall, the proportion of patients who continued to use aspirin, beta-blockers, ACEI/ARB, and statin therapy was 31.8%, 28.8%, 32.6%, and 24.7%, respectively. Significantly lower medication use was noted in older patients than in younger patients, including the use of aspirin (24.8% vs. 37.1%, p = 0.005), beta-blockers (20.3% vs. 34.8%, p = 0.001), ACEI/ARB (27.2% vs. 36.7%, p = 0.030), and statins (21.8% vs. 29.6%, p = 0.005).

The reasons for not taking the recommended medications varied between age groups (Table 2). More older patients complained about taking too many medications (48.7% vs. 17.9% for aspirin, 40.5% vs. 10.9% for beta-blocker, and 39.2% vs. 8.9% for ACEI/ARB) or considered medications to be non-essential (36.4% vs. 5.3% for statins, 32.7% vs. 12.6% for beta-blocker, and 37.8% vs. 7.7% for ACEI/ARB). Among younger patients, cost (37.8% vs. 10.9% for statin, 23.0% vs. 6.0% for beta-blocker, and 29.0% vs. 6.1% for ACEI/ARB) and fear of adverse effects (31.5% vs. 8.2% for aspirin, 37.4% vs. 7.7% for beta-blocker, and 43.2% vs. 7.4% for ACEI/ARB) were common reasons for not taking medications.

**Table 2:** **Reasons cited by patients who were not taking the recommended medications, by age group**

Reasons	65 years of age and older				Younger than 65 years			
	Aspirin	Statin	Beta-blocker	ACEI/ARB	Aspirin	Statin	Beta-blocker	ACEI/ARB
	n (%)							
Too expensive	11 (7.0)	18 (10.9)	10 (6.0)	9 (6.1)	21 (12.5)	71 (37.8)	40 (23)	49 (29.0)
Too many medications	77 (48.7)	47 (28.5)	68 (40.5)	58 (39.2)	30 (17.9)	18 (9.6)	19 (10.9)	15 (8.9)
Consider medications non-essential	32 (20.3)	60 (36.4)	55 (32.7)	56 (37.8)	28 (16.7)	10 (5.3)	22 (12.6)	13 (7.7)
Afraid of adverse effects	13 (8.2)	17 (10.3)	13 (7.7)	11 (7.4)	53 (31.5)	63 (33.5)	65 (37.4)	73 (43.2)
Side effects	16 (10.1)	14 (8.5)	15 (8.9)	8 (5.4)	16 (9.5)	16 (8.5)	20 (11.5)	12 (7.1)
Others	9 (5.7)	9 (5.5)	7 (4.2)	6 (4.1)	20 (11.9)	10 (5.3)	8 (4.6)	7 (4.1)

ACEI, angiotensin-converting enzyme inhibitor; ARB, angiotensin receptor blocker.

Overall, age was an independent predictor of medication underuse (OR = 1.05, 95% CI, 1.02 - 1.08, P = 0.002), even after adjusting for gender, education, income, insurance, comorbidities, PHQ scores, and the number of medications at discharge. In subgroups, low educational level, more comorbidities, and high numbers of discharge medications exhibited an independent and a positive impact on medication underuse in older patients, whereas low income and higher PHQ scores were predictors for medication underuse in younger patients (Table 3). Additionally, an inverse relationship between older age and recommended medication use was noted in both groups (Table 3).

**Table 3:** **Multivariate logistic regression models analysis for not taking any recommended medications at the time of the survey**

	65 years of age and older		Younger than 65 years	
	OR (95% CI)	p	OR (95% CI)	p
Age (per year)	1.12 (1.02 - 1.22)	0.015	1.11 (1.03-1.19)	0.008
Gender (vs. male)				
Female	0.32 (0.13 - 0.79)	0.013	0.95 (0.35 - 2.58)	0.921
Education (vs. senior high school or higher)				
Junior high school or lower	3.93 (1.65 - 9.32)	0.002	2.69 (0.86 - 8.46)	0.088
Income (vs. medium income or higher)				
Low income	0.58 (0.22 - 1.51)	0.264	3.97 (1.47 - 10.75)	0.007
Insurance (vs. basic medical insurance)				
Lack of insurance	2.65 (0.89 - 7.86)	0.080	2.58 (0.75 - 8.85)	0.131
Number of comorbidities	1.64 (1.12 - 2.39)	0.011	0.85 (0.39 - 1.84)	0.686
PHQ scores	0.97 (0.87 - 1.08)	0.570	2.62 (2.03 - 3.38)	< 0.001
Number of medications^*^	1.31 (1.11 - 1.55)	0.001	0.99 (0.70 - 1.42)	0.989
Number of outpatient cardiologist visits	1.16 (0.98 - 1.38)	0.086	1.02 (0.85 - 1.23)	0.822

^*^Number of prescribed medications at time of discharge; PHQ, Patient Health Questionnaire.

## Discussion

Overall, the proportions of ACS patients who continued to use aspirin, beta-blockers, ACEI/ARB, and statins at least one year after discharge were 31.8%, 28.8%, 32.6%, and 24.7%, respectively. Despite the fact that the number of prescribed medications at hospital discharge was high for the ACS patients, the rate of medication use reduced obviously one year after discharge. The treatment rate of recommended medications in the present survey is low compared with that employed in Western countries [9] or other surveys in mainland China [3, 20]. However, a comparison of these reports must be viewed with caution. Considerable differences exist between the present study and other studies, particularly with regard to the population selection criteria, ascertainment of medication use, and time points studied. Our study population consisted of a selected sample of consecutive ACS patients at least one year after hospital discharge, whereas the study subjects in other reports were selected from outpatient departments [3], which appear to underestimate the treatment rates of these medications because the patients who discontinued treatment may not visit the outpatient departments of hospitals. Additionally, in prior investigations, a subsequent ACS episode occurred in most study patients within 6 months to 1 year [20]. According to American Heart Association (AHA) guidelines, dual antiplatelet therapy consisting of aspirin and a thienopyridine agent, such as clopidogrel, should be administered for at least a full year and perhaps even longer in cases of certain stent placements in patients without a major risk for bleeding [21]. Thus, Studying consecutive patients may explain why the patients in our study, whose recent ACS episodes occurred after one year, were less likely to receive the recommended medical therapies.

In agreement with a previous report [16], the older patients in our study reported significantly reduced uses of recommended medications, even after adjusting for other co-factors, such as total number of discharge medications, education, and income. More importantly, this study identified age-related disparities in the factors associated with the underuse of recommended medications in CHD patients.

Low education level is correlated with medication underuse in older patients. This finding agrees with other studies demonstrating that low education and low health literacy are correlated with medication non-adherence [9]. Low educational status may indicate limited financial resources for medications or may reflect lower health literacy. Makaryus et al. reported that < 50% of patients could list all their medications and even fewer could recall the purpose of their medications at the time of hospital discharge [22]. Patients often delay filling prescriptions and have difficulty understanding medication regimens after hospital discharge [23]. Similarly, we observed that the majority of elderly patients considered their medications to be non-essential or that they lacked knowledge of the medications. Ineffective communication between the primary care physician and CHD patients can further compromise a patient’s understanding of his or her disease, its potential complications, and the importance of the prescribed medication [24]. Another possible explanation is that low education status may be associated with lower understanding of the benefits of continued medication use.

In the present study, cardiovascular-related comorbid conditions and other chronic illness were more prevalent in older patients. Conditions that are asymptomatic and chronic in nature and require long-term therapy are also associated with low medication use [9]. Older patients may have more severe diseases or comorbidities and were also more likely to be prescribed more evidence-based therapies. Polypharmacy continues to evolve, and new and synergic therapies for heart disease continue to be developed. A high number of concurrent medications were also a primary cause of medication underuse in elderly patients. Older patients more often reported not taking the recommended medications because of the large number of discharge medications. This result is consistent with a prior report indicating that as the total number of medications prescribed at the time of discharge increased, patient adherence to the cardiac regimen decreased [25]. This finding indicates that the complexity of the regimen can impact medication use. Thus, chronic disease management programs should consider all of the medications that patients are taking and reduce complexity by addressing multiple dosing frequencies whenever possible [26].

Among younger patients, high cost was an important reason for not taking medication. Cost issues have been identified as a major barrier to medication use [25, 27]. These analyses have demonstrated that disadvantaged financial status, as indicated by monthly incomes, was strongly associated with low medication use. Despite the rapid development of initiatives in mainland China to improve health insurance coverage [28], treatment costs are likely to remain an important predictor of low treatment rates. Considerable out-of-pocket medical costs are even encountered by patients with basic health insurance coverage and are an even great issue for patients with long-term treatments based on polypharmacy. The cost associated with hospitalization and discharge medications represents an unanticipated expense that can serve as a substantial burden, particularly for younger patients on fixed incomes. Furthermore, younger patients often have children and elderly parents to support and homes to maintain. Thus, chronic illnesses and medical expenditures could significantly reduce the financial resources of households [29]. It is unsurprising that even employed and insured patients are unable to keep up with increasing costs. Our findings reveal a major barrier for the medical care of patients at high risk for developing CHD in mainland China. Younger patients, particularly low-income patients with limited access to medical insurance, as defined in this study, represent a large portion of the population of China. Clearly, improvements in medical care for high-risk younger CHD patients in China will depend on increasing the affordability of essential medications, which could be addressed by either increased access to medical insurance, reduced medical care costs, or increased income for younger patients.

Depression is widely associated with heart disease. One in three ACS patients meet the criteria for minor depression [30]. We noted that younger patients with higher PHQ scores, indicative of depression, were associated with medication underuse. Specifically, the development of ischemic heart disease and related events may produce more dissatisfaction, sadness, and disappointment in a younger patient compared with an older patient who has become accustomed to chronic illness. Depression has been established as a risk factor for morbidity and mortality in CHD patients [31] and can substantially affect medication use [32], which is an important confounder that must be monitored and measured. Even mild depression is sufficient to dramatically alter compliance with essential therapy. A study of patients with ACS found that the nonadherence rate in patients without depression was 15% and increased to 30% in those with mild depression. Patients with moderate-to-severe depression were only slightly less adherent (37%), which suggests that the presence or absence of depression is far more important for medication use than the degree of depression [32, 33]. Based on these results, the mental health of patients contributes to the non-use of recommended medications. In contrast, depression improvements in cardiac patients were associated with improved medication use [34], indicating that the early diagnosis and treatment of depression is important in CHD patients.

Approximately 50% of the younger patients also reported being concerned about the adverse effects of their medications. The patients’ perceptions of adverse effects contributed significantly to their decisions regarding medication use. Previous studies have indicated that side effects are a common patient-reported reason for discontinuing the use of statin medications [35, 36]. A greater number of younger patients reported the discontinuation of beta-blockers due to feeling worse or not believing the medication was helping compared with the older age group. Various reports have indicated reduced adherence to beta-blockers use based on side effects, including sexual dysfunction [37], depression, and fatigue [38], which may be more notable in the younger group. Regardless of whether a patient’s perception of medication-induced side effects is objective and causal or a consequence of heightened awareness, it is important to address these concerns equally because patient perception, and not reality, leads to the underuse of recommended medications. Although younger patients in this study see physicians on a more regular basis, in an overtaxed health care system in which clinicians see a large volume of patients without resources to meet individual patient needs, the amount of time a clinician spends with a patient may be insufficient to properly assess and understand his medication-taking behaviors [24]. Thus, it is critical that adverse effect profiles are considered when prescribing medications and are discussed with the patient before the initial prescription and at every visit thereafter.

Several limitations must be considered in interpreting the results of the present study. The most significant limitation is that our study sample had a strong local geographical limitation. We also had a small study population, and our participants were recruited from a single center with a stable population. Although this factor may limit the generalizability of results to the wider Chinese population, the findings are likely to accurately reflect the situations within urban centers. Second, our study relied on patients’ self-reported medication use. Self-report measures can be biased by inaccurate patient recall or by social desirability, whereby patients report an overly optimistic estimation of medication use to their health providers. However, investigations that have used self-reporting to assess medication use have proven to be reliable and correlate well with pill count and electronic pill bottle monitoring [9, 39]. Third, we excluded very old patients from our study population because patients older than 85 years of age were substantially less likely to receive these recommended medications than younger patients. Indeed, specific evidence regarding the efficacy and cost-effectiveness of the study medications in very old patients is lacking. In general, old patients have been excluded from participating in all efficacy trials in which current clinical practice recommendations are based. Ultimately, the categorization of younger or older patients based on an age less than 65 years of age versus 65 years of age and older is arbitrary. Although there are commonly used definitions of old age, no general agreement on the age at which a person is considered old exists. Fourth, we cannot provide insight into post-discharge actions, such as prescribing recommended medications during a subsequent outpatient visit. However, a key message that emerges from a previous report is that the extent to which the medicine-taking behavior of individuals is influenced by physicians is substantial [40]. Existing research suggests that adherence to cardiac medications is improved by complete hospital discharge recommendations, particularly if physicians encourage their patients to obtain their medications and provide drug counseling [41, 42]. Fifth, we did not assess specific contraindications to medications; we only assessed the use of a specific medication if it was prescribed at discharge, suggesting that the patients were chronically taking the medications. Finally, although every effort was made to obtain information for all patients at the time of follow-up, information was available for only 81% of the patients. The limitations of this study must be considered when interpreting the results.

## Conclusions

At least one year after ACS hospital discharge, the underuse of medications in elderly patients is related to low education level, comorbidities, and a high total number of discharge medications; in younger patients, it is related to low-income levels and depression. The findings of the present study can be used to guide the development of age-related strategies to improve medication use for secondary prevention among CHD patients.

## Abbreviations

CHD: Coronary heart disease
ACS: Acute coronary syndrome
ACEI: Angiotensin-converting enzyme inhibitor
ARB: Angiotensin II receptor blocker
PHQ: Patient health questionnaire
AHA: American Heart Association.
